# Multidimensional Assessment of Patient-Reported-Outcomes in a Multicenter Cohort of Inborn Errors of Immunity

**DOI:** 10.1007/s10875-025-01972-1

**Published:** 2026-01-06

**Authors:** Melek Yorgun Altunbas, Asena Pinar Sefer, Sevgi Bilgic-Eltan, Cebbar Yildirimcakar, Pelin Ozturk, Ümmügülsüm Dikici, Ece Tüsüz Önata, Özge Atik, Hatice Eke-Gungor, Burcu Kolukisa, Hayrunnisa Bekis Bozkurt, Gaye Kocatepe, Şefika İlknur Kökcü Karadağ, Aysu İlhan Yalaki, Zeycan Canitez Oral, Nagihan Iskender, Tuğba Kıratlı Kıratlı Yolcu, Dilan Şirin, Reyhan Gumusburun, Demet Tekcan, Iknur Kulhas Celik, Salim Can, Razin Amirov, Necmiye Ozturk, Selcen Bozkurt, Burkay Cagan Colak, Ramin Mahmudov, Ezgi Yalcın Gungoren, Esra Karabiber, Hasibe Artac, Omur Ardeniz, Isıl Eser Simsek, Fatih Çelmeli, Tuğba Arikoglu, Deniz Özçeker, Dilara Kocacik Uygun, Aysen Bingol, Fatma Merve Tepetam, Öner Özdemir, Muhlis Cem Ar, Ebru Arik Yilmaz, Ahmet Ozen, Safa Baris, Elif Karakoc-Aydiner

**Affiliations:** 1 Department of Pediatric Allergy and Immunology, Faculty of Medicine, Marmara University, Istanbul, Turkey; 2 The Istanbul Jeffrey Modell Diagnostic Center for Primary Immunodeficiency Diseases, Istanbul, Turkey; 3 The Isil Berat Barlan Center for Translational Medicine, Istanbul, Turkey; 4 School of Medicine, Department of Pediatric Allergy and Immunology, Recep Tayyip Erdogan University, Rize, Turkey; 5 Division of Pediatric Allergy and Immunology, Pamukkale University Faculty of Medicine, Denizli, Turkey; 6 Department of Internal Medicine, Cerrahpasa Faculty of Medicine, Istanbul University-Cerrahpasa, Istanbul, Turkey; 7 Division of Allergy and Immunology, Department of Pediatrics, Research and Training Hospital of Sakarya University Medical Faculty, Sakarya, Turkey; 8 Department of Allergy and Immunology, Süreyyapaşa Chest Disease and Chest Surgery Training and Research Hospital, Health Sciences University, Istanbul, Turkey; 9 Department of Pediatric Allergy and Immunology, Health Sciences University, Kayseri City Education and Research Hospital, Kayseri, Turkey; 10 Van Education and Research Hospital, Pediatric Allergy and Immunology, University of Health Sciences, Van, Turkey; 11 Umraniye Research and Training Hospital, Pediatric Allergy and Immunology, University of Health Sciences, Istanbul, Turkey; 12 Department of Pediatric Allergy and Immunology, Akdeniz University Faculty of Medicine, Antalya, Turkey; 13 Department of Pediatric Allergy and Immunology, Prof. Dr. Cemil Tascıoglu City Hospital, Istanbul, Turkey; 14 Department of Pediatric Allergy and Immunology, Mersin University Faculty of Medicine, Mersin, Turkey; 15 Department of Pediatric Allergy and Immunology, University of Health Science, Antalya Education and Research Hospital, Antalya, Turkey; 16 Division of Pediatric Allergy and Immunology, Kocaeli University Faculty of Medicine, Kocaeli, Turkey; 17 Department of Internal Medicine, Division of Clinical Lmmunology and Allergy, Manisa Celal Bayar University Faculty of Medicine, Manisa, Turkey; 18 Department of Internal Medicine, Division of Allergy and Clinical Immunology, Ege University Medical Faculty, Izmir, Turkey; 19 Division of Pediatric Immunology and Allergy, Selcuk University Faculty of Medicine, Konya, Turkey; 20 Division of Adult Immunology and Allergy, Department of Chest Diseases, Marmara University, Pendik Training and Research Hospital, Istanbul, Turkey; 21 Division of Hematology, Department of Internal Medicine, Cerrahpasa Faculty of Medicine, Istanbul University-Cerrahpasa, Istanbul, Turkey

**Keywords:** Health-related Quality of Life, Immunoglobulin Replacement Therapy, Inborn Errors of Immunity, Patient-Centred Approach, Patient-Reported Outcomes, Treatment Satisfaction

## Abstract

Patient-reported outcomes are critical to multidisciplinary, patient-centred approaches in diseases requiring lifelong management. Among inborn errors of immunity (IEIs), reports on this subject are typically limited to specific diagnostic subgroups or focus narrowly on the route of immunoglobulin replacement therapy (IgRT), offering a restricted perspective. We aimed to evaluate the health-related quality of life (HRQoL) and IgRT-related treatment satisfaction (TS) of a heterogeneous cohort of IEI patients and identify factors influencing these outcomes to guide improving the health and well-being of IEI patients. We conducted a cross-sectional survey targeting IEI patients on IgRT, assessing TS (TSQM-9) and HRQoL (KINDL/SF-36). Patient/caregiver-reported data were integrated with clinical data to identify outcomes and influencing factors. The survey included 500 IEI patients (356 children, 144 adults) diagnosed 54% Primary Antibody Deficiency (PAD), 36% combined immunodeficiency, 7% immune-dysregulation, and 3% other IEIs. Non-PAD diagnoses, comorbidities, absence of school/work attendance, and IgRT-related systemic adverse reactions negatively impacted HRQoL. Severe infections and related hospitalizations adversely influenced both HRQoL and TS. The subcutaneous route of IgRT, particularly at home, was associated with higher TS due to its convenience and reduced school/work absenteeism. However, the IgRT route did not influence adult HRQoL. Patient-reported well-being and satisfaction in IEIs are multifactorial and cannot be solely attributed to the route of IgRT. Minimizing negative experiences related to the disease or its treatment and, where possible, encouraging patients to maintain school/work attendance or engage in activities that promote societal participation can enhance self-esteem, coping abilities, and overall well-being.

## Introduction

Inborn errors of immunity (IEIs) are rare hereditary disorders characterized by increased susceptibility to infections, allergic diseases, autoimmune conditions, inflammatory disorders, and malignancies [1]. Advances in next-generation sequencing have improved IEI diagnostics, led to novel gene discoveries, and deepened understanding of these disorders [2–4]. Enhanced diagnostic procedures and targeted therapies have extended patient survival [5–7]. However, frequent infections, hospitalizations, comorbidities, and treatment-related adverse effects remain challenges, significantly impacting patients' health-related quality of life (HRQoL) and overall well-being [8, 9]. These challenges, along with the chronic nature of the disease, significantly impair the social and physical functioning of IEI patients, negatively influencing their health-related quality of life (HRQoL) and well-being [10–14]

Immunoglobulin replacement therapy (IgRT), delivered intravenously (IVIG) or subcutaneously (SCIG), restores immunoglobulin (Ig) levels and reduces frequency and severity of infections in IEI patients [15–17]. IgRT is associated with high treatment satisfaction (TS)[18] which correlates with higher serum IgG trough levels, reduced infection frequency, fewer adverse reactions, and decreased injection site-related issues[19]. SCIG enables self-administration at home, but conventional 10% manual push requires weekly dosing and multiple infusion sites, potentially affecting quality of life and adherence[20, 21]. High-concentration (20%) SCIG and rHuPH20-facilitated SCIG (fSCIG) address these issues with less frequent dosing (every 2–4 weeks) and fewer infusion sites, improving convenience and adherence[22, 23]. Furthermore, research on the impact of IgRT methods on HRQoL and TS has predominantly been based on the transition from IVIG to SCIG, highlighting an improvement in HRQoL and TS compared to baseline [11, 24–26].

A recent bibliometric analysis and a systematic meta-analysis on HRQoL of IEIs emphasize not only the need to incorporate HRQoL parameters into the patient-centred, multidisciplinary, and holistic approach recommended for their overall health assessment and management but also highlights the inadequacy of comprehensive HRQoL evaluations in the current IEI literature [27, 28].

We aimed to comprehensively evaluate HRQoL, TS related to IgRT, and the factors influencing these outcomes in a large and diverse cohort of IEI patients and guide researchers focusing on IEIs and clinicians managing these patients.

## Materials and Methods

### Study Population

In the study, patients with IEI from seventeen medical centers were enrolled. Diagnosis and classifications were determined according to the International Union of Immunological Societies and the Middle East and North Africa Diagnosis and Management Guidelines [1, 29]. We included patients who had been receiving IgRT for at least one year, had not experienced any changes in the route of IgRT administration during the past year, and had no active infection at the time of evaluation. Patients who did not provide informed consent were excluded from the study.

### Data Collection

The demographic, clinical, and laboratory data of the patients were obtained from electronic medical records and written patient files, including age, sex, diagnosis, age at symptom onset and age at diagnosis, comorbidities, the frequency of infections diagnosed and treated by a physician, the frequency of prescribed antibiotics, the number of days hospitalized due to infections, and the number of school/work absenteeism days, all within the last year. Comorbidities were categorized based on the affected systems: bronchiectasis and chronic lung disease as pulmonary; congenital heart disease and heart failure as cardiac; mental/motor retardation, epilepsy, and cerebrovascular events as neurological; diabetes, thyroiditis, and osteoporosis as endocrinological; liver failure and hepatitis as hepatic; severe eczema, alopecia, and vitiligo as dermatological; inflammatory bowel disease and celiac disease as enteropathy, rheumatoid arthritis, Sjogren's syndrome, and seronegative arthritis as rheumatological; malignancy, lymphoproliferation, and cytopenia as hematological/oncological; and asthma, food, and drug allergies as allergic comorbidities.

The infections were categorized and recorded as serious bacterial infections (SBIs) and non-serious infections (NSIs). The SBIs were recorded as defined by the Food and Drug Administration Guidance for Industry on Intravenous Immunoglobulin [30] and the European Medicines Agency guidelines [31], including microbiologically and/or radiologically confirmed bacteremia, sepsis, bacterial meningitis, osteomyelitis/septic arthritis, bacterial pneumonia, and visceral abscess. Data on IgRT were recorded, including the administration route (intravenous or subcutaneous), concentration (5%, 10%, 20%), dosage, frequency (in days), duration (in hours), use of an infusion pump, IgRT-related local and systemic adverse reactions (AR), and serum trough (for IVIG) or steady-state (for SCIG) IgG levels.

### Questionnaires for Health-Related Quality of Life and Treatment Satisfaction

HRQoL was evaluated by using both the Kinder Lebensqualitätsfragebogen: Children's Quality of Life Questionnaire (KINDL)-Child survey for the patients aged 4–18 years and relevant KINDL-Parent questionnaires, previously validated for Turkish children [32]. For child participants with intellectual disability or those unable to comply with the questionnaire, only the KINDL-Parent questionnaire was administered to assess their quality of life.

KINDL is a self-report questionnaire available for three different age groups: Kiddy-KINDL (for aged 4–7 years), Kid-KINDL (8–12 years), and Kiddo-KINDL (for adolescents). Additionally, there are two KINDL-Parent versions: one for children (ages 3–7) and one for adolescents (ages 8–18) from the parental perspective. The KINDL questionnaires consist of seven subscales: physical health, emotional health, self-esteem, family, friends, daily functioning (school or preschool/kindergarten), and disease. Scores from these seven subscales were transformed into values ranging from 0 to 100, with higher scores indicating better life quality [32–34].

The HRQoL in adult participants was assessed using the Short Form Health Survey (SF-36) questionnaire. The SF-36 questionnaire comprises 36 items that measure eight domains of health concepts: general health perceptions, physical functioning, physical role limitations, social functioning, mental health, bodily pain, vitality (energy/fatigue), and emotional role limitations. The SF-36 is scored on a scale from 0 to 100, with higher scores representing better functionality and well-being [35].

All adult and pediatric participants' treatment satisfaction was assessed by administering the Treatment Satisfaction Questionnaire for Medication-9 (TSQM-9) to the patients or parents. The TSQM-9 questionnaire was administered to the parents of patients younger than 12 years of age or those with intellectual disability. All other patients answered the questionnaire themselves. The TSQM-9 questionnaire consisted of 9 questions, organized into 3 subscales: effectiveness (questions 1–3), convenience (questions 4–6), and global satisfaction (questions 7–9). The total score is derived from these 9 questions. TSQM-9 domain scores range from 0 to 100, with higher scores indicating greater satisfaction [36].

### Statistics

Statistical analyses were conducted by Jamovi 2.3.26 version (The Jamovi Project, Australia). Results are presented as medians and interquartile ranges (IQR 25th-75th percentiles) due to the non-normal distribution. Continuous variables between two groups were compared using the Mann–Whitney U test, and comparisons among three groups were made using the Kruskal–Wallis test, followed by post-hoc pairwise comparisons with Bonferroni correction to adjust for multiple testing. The categorical variables between groups were compared using the chi-square test. A* p*-value below 0.05 was considered statistically significant. For analytical consistency, IgRT doses were standardized to a 21-day equivalent during statistical analysis only; this adjustment did not involve any modifications to patients’ actual treatment regimens. Graphs were produced using GraphPad Prism 10.4.1 (GraphPad Software Inc., San Diego, California).

## Results

### Patient Characteristics

**Fig. 1:**
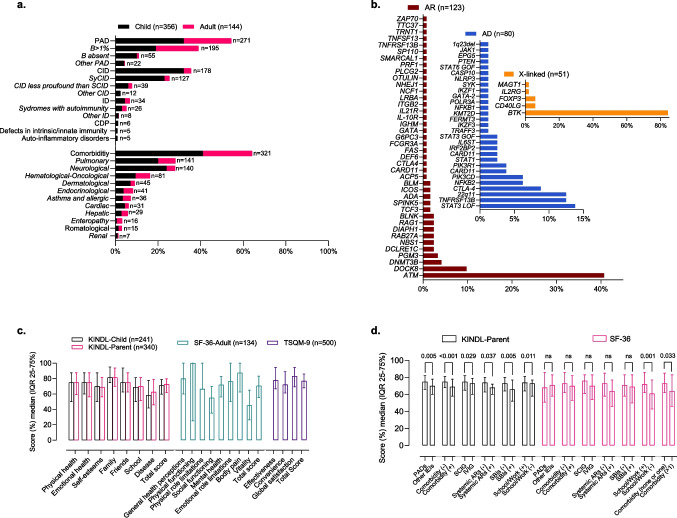
**A)** Clinical characteristics of IEI patients **B)** Characteristics of Genetic Defects in Genetically Diagnosed IEI Patients C**)** KINDL-Child, KINDL-Parent, SF-36, and TSQM-9 total and subscale scores **D)** Comparison of KINDL-Parent and SF-36 total scores between groups. The scores are presented as median (IQR 25–75%), Mann–Whitney U test, *p < *0.005, statistically significant. AD, autosomal dominant; AR, autosomal recessive; ARs, adverse reactions; CDP, congenital defects of phagocyte; CID, combined immunodeficiency; ID, immune dysregulation; IEI, inborn errors of immunity; IQR, interquartile range; IVIG, intravenous immunoglobulin; KINDL, Kinder Lebensqualitätsfragebogen: Children's Quality of Life Questionnaire; PAD, predominantly antibody deficiency; SBI, serious bacterial infection; SCIG, subcutaneous immunoglobulin; SF-36, Short Form Health Survey-36; SyCID, syndromic combined immunodeficiency; TSQM-9, Treatment Satisfaction Questionnaire for Medication-9

A total of 500 patients were included in the study. The demographic and clinical characteristics of the participants are presented in detail in Table 1, Fig. 1 A and B.

**Table 1:** Demographics and Clinical Characteristics of Patients with Inborn Errors of Immunity

	All n = 500 (100%)	Child n = 356 (100%)	Adult n = 144 (100%)
Sex, n (%)			
Male	294 (59%)	204 (57%)	90 (63%)
Female	206 (41%)	152 (43%)	54 (37%)
Age (years), median (IQR; 25–75)	13 (7–20)	9 (5–12)	31 (22–42)
Age at symptom onset (years), median (IQR; 25–75)	1 (0.5–4)	1 (0–2)	6 (1–15)
Age at diagnosis (years), median (IQR; 25–75)	5 (2–10)	3 (1–7)	17 (8–30)
Diagnostic delay (years), median (IQR; 25–75)	2 (1–6)	1.5 (0.5–4)	5 (2–12)
≤ 2 years, n (%)	259 (52%)	220 (62%)	39 (27)
> 2 year, n (%)	241 (48%)	136 (38%)	105 (73)
Diagnosis, n (%)			
PAD	272 (54%)	160 (45%)	112 (78)
CID	178 (36%)	158 (44%)	20 (14%)
ID	34 (7%)	22 (6%)	12 (8%)
CDP	6 (1%)	6 (2%)	-
Defects in intrinsic and innate immunity	5 (1%)	5 (1.5%)	-
Auto-inflammatory disorders	5 (1%)	5 (1.5%)	-
Genetically Diagnosed, n (%)	254 (51%)	218 (85%)	36 (25%)
AR	123 (25%)	115 (32%)	8 (6%)
AD	80 (16%)	58 (16%)	22 (15%)
X-linked	51 (10%)	45 (13%)	6 (4%)
Comorbidity presence, n (%)	306 (65%)	206 (58%)	115 (80%)
IgRT administration Route, n (%)			
IVIG (at the hospital)	363 (73%)	264 (74%)	99 (69%)
SCIG (at home)	137 (27%)	98 (26%)	45(31%)
SCIG 20% (pump-assisted)	71 (14%)	51 (14%)	20 (14%)
fSCIG 10% (pump-assisted)	44 (9%)	28 (8%)	16 (11%)
SCIG 10% Conventional, manual)	22 (4%)	13 (4%)	9 (6%)
IgRT- related adverse reactions, n (%)	144 (29%)	88 (25%)	56 (39%)
Systemic, n (%)	97 (19%)	60 (17%)	37 (26%)
Local, n (%)	54 (11%)	30 (8%)	24 (17%)
Serum IgG (mg/dl), median (IQR; 25–75%)	917 (772–1171)	912 (742–1173)	953 (743–1170)
^‡^Number of Infections, median (IQR; 25–75%)	2 (1–4)	2 (0–3)	2 (1–4)
^‡^Number of antibiotics prescribed	1 (0–3)	1 (0–3)	1 (0–3)
^‡^Number of SBIs	0 (0–0)	0 (0–0)	0 (0–0)
^‡^Number of Hospitalisation days	0 (0–0)	0 (0–1)	0 (0–0)
School/work attendance, n (%)	304 (61%)	212 (60%)	92 (64%)
^‡^School/work absence days, median (IQR; 25–75%)	17 (8–25)	15 (10–25)	17 (5–30)
KINDL-Child respondent, n (%)	241 (48%)	241 (68%)	-
KINDL-Parent respondent, n (%)	340 (68%)	340 (96%)	-
SF-36 respondent, n (%)	134 (27%)	-	134 (93%)
TSQM-9 respondent, n (%)	500 (100%)	356 (100%)	144 (100%)

AD, autosomal dominant; AR, autosomal recessive; CDP, congenital defects of phagocyte; CID, combined immunodeficiency; fSCIG, facilitated subcutaneous immunoglobulin; ID, immune dysregulation; IgRT, immunoglobulin replacement therapy; IQR, interquartile range; IVIG, intravenous immunoglobulin; KINDL, Kinder Lebensqualitätsfragebogen: Children's Quality of Life Questionnaire; PAD, predominantly antibody deficiency; SBI, serious bacterial infection; SCIG, subcutaneous immunoglobulin; SF-36, Short Form Health Survey-36; TSQM-9, Treatment Satisfaction Questionnaire for Medication-9. ^‡^, in the last one year

### Health-Related Quality of Life and Treatment Satisfaction Surveys

TS was evaluated in all patients (100%), while HRQoL was assessed in 95% of the participants.

HRQoL assessment was not feasible in participants younger than three years of age, who constituted 4% of the pediatric patients, due to the lack of an age-appropriate questionnaire. Out of the 144 adult patients, 7% were unable to respond to the SF-36 questionnaire due to intellectual disability. In assessing HRQoL in children, the KINDL-Parent survey was completed by 96% of parents, and the KINDL-Child version by 68% of children.

The median total score for the KINDL-Child was 71, with an IQR of 60 to 79, while the KINDL-Parent reflected a median score of 73 (IQR 62–80). Within the subscales of KINDL, the disease subscale yielded the lowest scores for both KINDL-Child (median 58, IQR 42–78) and KINDL-Parent (median 63, IQR 46–79). Conversely, the highest scores were recorded in the family subscale, with a median of 81.3(IQR 75–95) for children and 81 (IQR 70–94) for parents, indicating consistency across respondents (Fig. 1C). Among 241 pediatric patients who responded to both the parent and child questionnaires, the median total score for the KINDL-Parent was significantly higher at 73 (IQR 64–81) compared to the KINDL-Child at 71 (IQR 60–79) (*p =* 0.008). When analysing which subscale contributed to the higher scores, it was found that the disease subscale had a significantly higher median score in parent-reported outcomes (67, IQR 53–79) compared to child-reported outcomes (58, IQR 42–78) (*p < *0.001). No significant differences were found in the other subscales. Consequently, the KINDL-Parent scores were adopted as the primary measure for evaluating HRQoL in children, given that 96% responded to the KINDL-Parent survey.

In adult IEIs, the SF-36 survey revealed a median total score of 71 (IQR 55–83). The general health perceptions subscale scored the lowest with a median of 45 (IQR 26–65), while the highest median was observed in the physical role limitations subscale with a median of 100 (IQR 25–100) (Fig. 1C).

The total score of TSQM-9 showed a median of 77 (IQR 68–86). Effectiveness subscale presented a median of 78 (IQR 68–94), convenience subscale a median of 72 (IQR 61–89), and global satisfaction subscale a median of 83 (IQR 64–94) (Fig. 1C).

### Comparison of HRQoL and TS between groups

Patients were divided into two groups based on sex, age, diagnosis (PADs or other IEIs), diagnostic delay (≤ 2 years or > 2 years), comorbidities, IgRT-related systemic AR, SIBs, hospitalization due to infections, IgRT route (IVIG/SCIG), and school/work attendance. Higher HRQoL was significantly associated with PAD diagnosis, absence of comorbidities, lack of IgRT-related systemic ARs or any SIBs, SCIG route of IgRT, and presence of school or work attendance (*p =* 0.026, *p < *0.001, *p =* 0.005, *p =* 0.015, *p =* 0.029, *p < *0.001 respectively). IVIG as the method of IgRT, presence of at least one SBI in the last year, or at least one hospitalization due to infection were found to be statistically significantly associated with lower TSQM-9 scores (*p =* 0.022, *p =* 0.001, *p =* 0.002, respectively), as depicted in Table 2.

**Table 2:** Factors Influencing Health-Related Quality of Life and Treatment Satisfaction in Patients with Inborn Errors of Immunity

	HRQoL Total Score %			TSQM-9 Total Score %		
	n = 474 (100%)	Median (IQR; 25–75%)	*P* *value*	n = 500 (100%)	Median (IQR; 25–75%)	*p value*
**Sex**						
**Male**	281 (43%)	73 (61–86)	*0.078*	294 (59%)	78 (70–86)	*0.145*
**Female**	193 (57%)	71 (58–80)		206 (41%)	76 (66–86)	
**Age (years)**						
< 18	340 (72%)	73 (62–80)	*0.654*	356 (71%)	76 (68–86)	*0.289*
≥ 18	134 (28%)	71 (55–83)		144 (29%)	80 (66–92)	
**Diagnosis**						
PADs	262 (55%)	74 (62–82)	0.026*	272 (54%)	78 (68–90)	*0.083*
Other IEIs	212 (45%)	70 (59–59)		228 (46%)	76 (68–84)	
**Comorbidity**						
presence	306 (65%)	69 (57–79)	< 0.001*	321 (64%)	76 (68–88)	*0.591*
absence	168 (35%)	75 (66–82)		179 (36%)	78 (68–86)	
**Neurological Comorbidity**						
presence	128 (27%)	67 (57–77)	< 0.001*	140 (28%)	76(68–85)	*0.348*
absence	346 (73%)	74 (63–82)		360 (72%)	78 (68–88)	
**Haematological/Oncological Comorbidity**						
presence	80 (17%)	68 (54–78)	0.033*	81 (16%)	80 (70–86)	*0.291*
absence	394 (83%)	73 (61–81)		419 (84%)	76 (67–87)	
**Diagnostic delay**						
> 2 years	318 (67%)	72 (58–82)	*0.441*	328 (66%)	76 (66–88)	*0.941*
≤ 2 years	156 (33%)	73 (63–80)		172 (34%)	78 (70–86)	
**IgRT route**						
IVIG	342 (72%)	71 (58–99)	0.029*	363 (73%)	76 (68–86)	0.022*
SCIG	132 (28%)	75 (65–82)		137 (27%)	82 (70–88)	
**IgRT-related Systemic AR**						
presence	91 (19%)	68 (56–77)	0.005*	97 (19%)	76(64–88)	*0.440*
absence	383 (81%)	74 (61–81)		403 (81%)	78(68–86)	
^**‡**^**Hospitalization**						
presence	105 (22%)	70 (57–78)	*0.068*	114 (23%)	74 (66–82)	0.002*
absence	369 (78%)	73 (62–81)		386 (77%)	78 (66–88)	
^**‡**^**SBI**						
presence	72 (15%)	66 (52–73)	0.015*	78 (16%)	72 (62–80)	0.001*
absence	402 (85%)	73 (62–81)		422 (84%)	78(68–88)	
**School/work attendance**						
presence	303 (64%)	74 (63–82)	0.001*	304 (61%)	77 (68–88)	*0.470*
absence	171 (36%)	69 (57–77)		196 (39%)	77 (68–85)	

AR, adverse reaction; HRQoL, health-related quality of life; IEI, inborn errors of immunity; IgRT, immunoglobulin replacement therapy; IQR, interquartile range; IVIG, intravenous immunoglobulin; PAD, predominantly antibody deficiency; SBI, serious bacterial infection, SCIG, subcutaneous immunoglobulin; ^‡^, in the last one year; *p < 0.005, Mann–Whitney U test

When the group comparisons that showed statistically significant differences in HRQoL scores were applied separately to the KINDL-Parent and SF-36 total scores, the results revealed the following: Significantly higher total scores were observed in the KINDL-Parent survey across all comparisons whereas, for SF-36, only individuals with school/work attendance had significantly higher median scores than those without (Fig. 1D). But, considering that 80% of adult patients have at least one comorbidity, when we compared the SF-36 scores between those with multiple comorbidities and those without, the median total score in the group with multiple comorbidities (64, IQR; 46–83) was significantly lower than in the group without multiple comorbidities (73, IQR; 62–85) (*p =* 0.033) (Fig. 1D).

### Subscales of HRQoL and TS

**Fig. 2:**
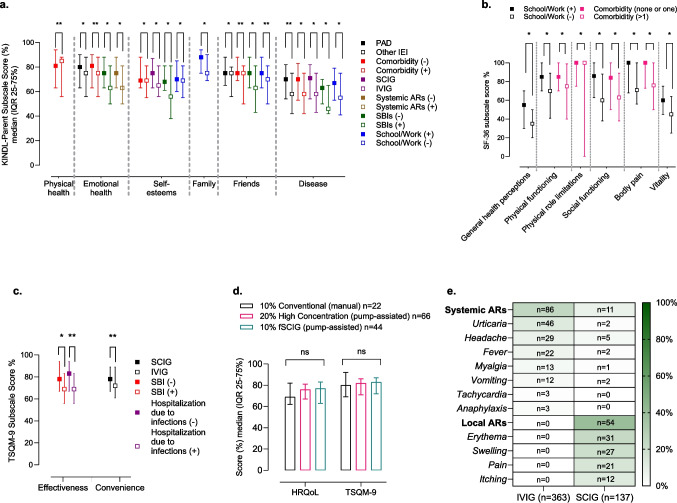
**A)** Comparison of KINDL-Parent subscale scores between groups **B)** Comparison of SF-36 Subscale Scores between groups **C)** Comparison of TSQM-9 subscale scores between groups **D)** Comparison of HRQoL and TSQM-9 total scores by SCIG administration method **E)** Detailed characteristics of IgRT-related adverse reactions in IVIG and SCIG groups. The scores are presented as median (IQR 25–75%), Mann–Whitney U test, Kruskal–Wallis tests (followed by post-hoc pairwise comparisons with Bonferroni correction to adjust for multiple testing), *p < *0.005, statistically significant, *p < 0.005, **p < 0.001. ARs, adverse reactions; HRQoL, health-related quality of life; fSCIG, facilitated subcutaneous immunoglobulin; IEI, inborn errors of immunity; IgRT, immunoglobulin replacement therapy; IQR, interquartile range; IVIG, intravenous immunoglobulin; KINDL, Kinder Lebensqualitätsfragebogen: Children’s Quality of Life Questionnaire; ns, not significant; SBI, serious bacterial infection; SCIG, subcutaneous immunoglobulin; SF-36, Short Form Health Survey-36; TSQM-9, Treatment Satisfaction Questionnaire for Medication-9

Further subgroup comparisons were conducted to investigate the specific subscales contributing to the observed differences in HRQoL. The KINDL-Parent subscale scores revealed significant findings across multiple categories. In the comparison between PADs and other IEIs, higher scores were observed in the PAD group for emotional health (*p =* 0.011), friends (*p =* 0.019), and disease (*p =* 0.030) subscales. Children without comorbidities scored significantly higher in physical health (*p < *0.001), emotional health (*p < *0.001), self-esteem (*p =* 0.012), friendships (*p < *0.001), and disease (*p =* 0.004) subscales. A comparison of IVIG and SCIG groups showed that SCIG patients had higher scores for self-esteem (*p =* 0.009) and disease (*p =* 0.004) subscales. For patients without systemic ARs, the emotional health subscale scores were higher than those with (*p =* 0.002), and without SBI had significantly higher scores for emotional health (*p =* 0.011), self-esteem (*p =* 0.005), friends (*p =* 0.015), and disease (*p =* 0.002) subscales. Lastly, school or work attendance was associated with higher scores for the self-esteem (*p =* 0.026), family (*p =* 0.027), friends (*p < *0.001), and disease (*p =* 0.001) subscales, as depicted in Fig. 2A.

The SF-36 subscale scores revealed the following significant differences between patients with and without school/work attendance: physical (*p =* 0.003), vitality (*p =* 0.005), mental health (*p =* 0.008), social functioning (*p =* 0.011), bodily pain (*p =* 0.008) and general health perceptions (*p =* 0.002). When comparing the SF-36 subscale scores between the groups with and without multiple comorbidities, the group without multiple comorbidities showed significantly higher scores in the following subscales: physical function, physical role limitation, social function, and bodily pain (*p =* 0.003, *p =* 0.001, *p =* 0.041, *p =* 0.010, respectively) (Fig. 2B).

Analysis of subscale contributions to TSQM-9 total score differences among specific groups revealed the convenience subscale score was significantly higher in the SCIG group than in the IVIG group (*p =* 0.021). The effectiveness subscale score was higher in patients without SBIs than in those with (*p < *0.001), and in participants without hospitalizations due to infections compared to those with (*p < *0.001) (Fig. 2C).

In the comparison of HRQoL and TSQM-9 total scores between conventional (10%) SCIG, High Concentration (20%) SCIG, and fSCIG groups, no significant difference was found between the three groups in HRQoL and TSQM-9 scores (*p =* 0.742, *p =* 0.847, respectively) (Fig. 2D).

To investigate the reasons behind the higher HRQoL and TSQM-9 scores observed in the SCIG group compared to the IVIG group, we compared the IgRT dose, trough/stable serum IgG levels (mg/dL), the total number of infections, the number of prescribed antibiotics, the presence and frequency of SIBs and NSIs, the presence of hospitalizations and number of hospitalizations days, the number of school/work absentee days, and the presence of IgRT-related systemic or local ARs between the two groups. Among all these variables, only the number of school/work absenteeism days and IgRT-related systemic and local ARs showed a significant difference between the two groups (*p < *0.001, *p < *0.001, *p < *0.001, respectively) **(**Table 3**).** Characteristics of systemic and local ARs of SCIG and IVIG groups are detailed in Fig. 2E.

**Table 3:** Comparison of Variables Between IVIG and SCIG Groups

	**IVIG** **n = 363 (100%)**	**SCIG n = 137(100%)**	***p*** ***value***
^**┼**^IgRT dose (g/kg), median (IQR; 25–75%)	0.46 (0.37–0.5)	0.38 (0.33–0.44)	< 0.001*
Serum IgG level (mg/dl), median (IQR; 25–75%)	910 (750–1151)	936 (698–1200)	*0.97*
Number of Infections, median (IQR; 25–75%)	2 (0–4)	2 (1–3)	*0.411*
^**‡**^SBI presence, n (%)	63 (17%)	15 (11%)	*0.078*
^**‡**^Number of SBIs, median (IQR; 25–75%)	0 (0–0)	0 (0–0)	*0.084*
^**‡**^Number of NSIs, median (IQR; 25–75%)	2 (0–3)	2 (1–3)	*0.0584*
^**‡**^Number of antibiotics prescribed, median (IQR; 25–75%)	1 (0–3)	1 (0–2)	*0.409*
^**‡**^Hospitalization presence, n (%)	88 (24%)	26 (19%)	*0.211*
IgRT-related Systemic adverse reaction presence, n (%)	86 (24%)	11 (8%)	< 0.001*
IgRT-related Local adverse reaction presence, n (%)	0 (0%)	54 (39%)	< 0.001*
^**‡**^School/work absentee days, median (IQR; 25–75%)	20 (14–30)	7 (2–12)	< 0.001*

IgG; immunoglobulin G; IgRT, immunoglobulin replacement therapy; IQR, interquartile range; IVIG, intravenous immunoglobulin; NSI, nonserious infections; SBI, serious bacterial infection, SCIG, subcutaneous immunoglobulin; ^**‡**^**,** in the last one year; ^**┼**^, The doses were standardized to 21-day intervals to ensure comparability across treatment schedules; ^**‡**^ in the last one year; *p < 0.005**,** Mann–Whitney U test, Chi-square test

## Discussion

The current study identified factors associated with better HRQoL and satisfaction with IgRT therapy from a multidimensional perspective in a large and heterogeneous cohort that accurately represents the IEI population in routine clinical practice. The relatively low total HRQoL scores and low general health perception domain observed in our IEIs cohort support previous reports [37–40], with low scores generally associated with disease severity and negative experiences, including diagnoses other than PAD, comorbidities, SBIs, and IgRT-related systemic AR, while better HRQoL was found to be associated with school/work attendance and receiving SCIG therapy at home. Better IgRT-related TS was associated with receiving SCIG at home and the absence of challenges that could raise concerns about treatment efficacy, such as experiencing severe bacterial infections and hospitalizations due to infections.

A significant proportion of the non-PAD group in our study consisted of participants with a primary diagnosis of CID, a condition characterized by a more severe clinical course and poorer survival outcomes relative to PADs [41]. The observation that patients diagnosed with PAD report better HRQoL compared to those with other IEIs demonstrates that the milder clinical course of PADs compared to CIDs[42] is reflected in patient-reported outcomes.

Although the negative impact of multiple chronic conditions on patient-reported outcomes is well-documented [43], this topic has been addressed in only a limited number of small cohorts of IEIs[10, 44]. In our large cohort, which supports existing knowledge while also providing detailed insights specific to IEIs, the presence of comorbidities, regardless of their number, negatively affected HRQoL in children but had no significant impact on adult participants. However, in adults, the presence of multiple simultaneous comorbidities was found to be associated with reduced HRQoL. Notably, the majority of adult patients in our cohort were diagnosed with PADs (78%) and had at least one comorbidity (80%). The high prevalence of PAD diagnosis and comorbid conditions in adults explains why the diagnostic subgroup (PAD or other IEIs) and the mere presence of comorbidity did not significantly influence HRQoL, but the cumulative burden of multiple comorbidities had a pronounced negative effect.

It should be considered that comorbidity, non-PAD diagnoses, and SBIs in pediatric IEIs affect not only physical well-being but also emotional, social, and self-esteem domains and it is known that low self-esteem can negatively impact physical and mental health as well as coping behaviors[45, 46]. In this context, focusing solely on the physical health of these patients may be inadequate for management. Integrating social and emotional support into medical care, in proportion to the number and severity of clinical challenges requiring coping strategies, can significantly contribute to overall well-being. Our findings indicate that receiving IgRT as SCIG at home and maintaining school attendance positively influence the physical, emotional, and self-esteem domains of HRQoL. This result aligns with the concept that in children, parental and peer approval, as well as participation and competence in academic or other activities, contribute to increased self-esteem [47]. Based on these, promoting home-based SCIG therapy as the preferred IgRT method for patients with frequent clinical challenges, and encouraging participation in school or similar activities to the extent that physical conditions allow, would be a meaningful effort to enhance relevant aspects of HRQoL and facilitate coping.

Our findings, demonstrating that multiple comorbidities and unemployment negatively impact both the physical and social domains of HRQoL in adults with IEIs, support previous reports in the context of chronic diseases in general [48, 49], and with a small cohort of patients diagnosed with PADs[10] Additionally, the significantly lower scores in the physical role limitation domain observed in adults with multiple comorbidities suggest that a comprehensive assessment of patient-reported outcomes could serve as a preliminary evaluation for identifying comorbidities contributing to physical limitations.

Studies investigating the relationship between IgRT therapy and HRQoL often highlight improvements in HRQoL following the switch from IVIG to SCIG, demonstrating that these improvements are associated with increases in general health perception and family relationship subscales [50–55]. Although our study provides a cross-sectional evaluation of HRQoL, the observed association between SCIG and better HRQoL and its positive impact on the general health perception subdomain in children supports existing evidence. However, the finding of SCIG's positive effect on self-esteem represents a novel outcome that diverges from previous reports in this area [11, 26, 56].

Home-based IgRT therapies, regardless of the administration route (intravenous or subcutaneous), are known to be associated with higher HRQoL, TS, and preference [18]. SCIG treatment is favored due to its lower systemic side effects, reduced school and work absenteeism, steady-state IgG levels, and ease of self-administration compared to IVIG [11, 18, 25, 51, 57–59]. Also, a recent study demonstrated that SCIG at home was associated with higher convenience and effectiveness than IVIG. High effectiveness was reported to be achieved by serum steady-state IgG levels [60] The absence of differences in IgG levels, infection rates, and effectiveness subscores of TSQM-9 between the SCIG and IVIG and the similar score of HRQoL in two groups of adults, contradict previous reports. However, prior studies primarily focused on patients switching from IVIG to SCIG due to treatment dissatisfaction [11, 24–26]. Such findings are likely subject to bias and may not accurately reflect real-world experiences. In contrast, we conducted an observational study in an intervention-free setting, analysing patient-reported outcomes cross-sectionally in patients who had not switched IgRT methods for at least one year. The differences in results are likely related to the real-life experience reflected in the current study design. However, when comparing conventional (10%) SCIG, high-concentration (20%) SCIG, and fSCIG, we found similar HRQoL and TS across all three groups. This suggests that the reported benefits of IgRT, as perceived by patients, are primarily attributable to shared factors among these SCIG methods, including the convenience of home administration, reduced school/work absenteeism, and fewer systemic adverse reactions.

In conclusion, patient-reported outcomes in IEIs are influenced by multiple factors, including disease severity, comorbidities leading to physical limitations, severe infections despite treatment, and side effects of the therapy. These outcomes are crucial in the patient-centred assessment of these patients. Prevention and effective management of comorbidities, minimizing infections, and supporting patient independence are essential components of IEI patient care. To the extent permitted by their physical condition, adults should be encouraged to work, and children to attend school, participate in special education programs, or engage in activities that promote a sense of competence. Encouraging parents to support these efforts should also be an integral part of patient-centred management. The route of IgRT is not always a primary factor in treatment satisfaction for IEI patients. Evaluating individual biological IgG levels to prevent severe infections and hospitalizations on a patient-specific basis can enhance IgRT-related treatment satisfaction in the management of IEI patients.

## Data Availability

The datasets generated during and/or analysed during the current study are available from the corresponding author on reasonable request.
